# Identification of cyclin B1 and Sec62 as biomarkers for recurrence in patients with HBV-related hepatocellular carcinoma after surgical resection

**DOI:** 10.1186/1476-4598-11-39

**Published:** 2012-06-08

**Authors:** Li Weng, Juan Du, Qinghui Zhou, Binbin Cheng, Jun Li, Denghai Zhang, Changquan Ling

**Affiliations:** 1 Department of Traditional Chinese Medicine, Changhai Hospital, Second Military Medical University, Shanghai, 200433, People’s Republic of China; 2 Gongli Hospital, Shanghai, 200135, People’s Republic of China; 3 Department of Traditional Chinese Medicine, Changhai Hospital, Second Military Medical University, 168 Changhai Road, Shanghai, 200433, People’s Republic of China

**Keywords:** Hepatocellular carcinoma, Recurrence, Cyclin B1, Sec62, Birc3

## Abstract

**Background:**

Hepatocellular carcinoma (HCC) is the fifth most common cancer worldwide. Frequent tumor recurrence after surgery is related to its poor prognosis. Although gene expression signatures have been associated with outcome, the molecular basis of HCC recurrence is not fully understood, and there is no method to predict recurrence using peripheral blood mononuclear cells (PBMCs), which can be easily obtained for recurrence prediction in the clinical setting.

**Methods:**

According to the microarray analysis results, we constructed a co-expression network using the k-core algorithm to determine which genes play pivotal roles in the recurrence of HCC associated with the hepatitis B virus (HBV) infection. Furthermore, we evaluated the mRNA and protein expressions in the PBMCs from 80 patients with or without recurrence and 30 healthy subjects. The stability of the signatures was determined in HCC tissues from the same 80 patients. Data analysis included ROC analysis, correlation analysis, log-lank tests, and Cox modeling to identify independent predictors of tumor recurrence.

**Results:**

The tumor-associated proteins cyclin B1, Sec62, and Birc3 were highly expressed in a subset of samples of recurrent HCC; cyclin B1, Sec62, and Birc3 positivity was observed in 80%, 65.7%, and 54.2% of the samples, respectively. The Kaplan-Meier analysis revealed that high expression levels of these proteins was associated with significantly reduced recurrence-free survival. Cox proportional hazards model analysis revealed that cyclin B1 (hazard ratio [HR], 4.762; *p* = 0.002) and Sec62 (HR, 2.674; *p* = 0.018) were independent predictors of HCC recurrence.

**Conclusion:**

These results revealed that cyclin B1 and Sec62 may be candidate biomarkers and potential therapeutic targets for HBV-related HCC recurrence after surgery.

## Background

Hepatocellular carcinoma (HCC) is the fifth most frequent cancer and the third leading cause of cancer-related deaths worldwide, with over a half million deaths per annum[1]. The annual incidence of HCC in hepatitis B cirrhotic patients can run as high as 3–5%, and one-third will develop HCC in their lifetime [2]. In China, an endemic area with almost one third of the HBsAg carriers found worldwide [3]. Because of high infection rates with hepatitis B virus (HBV), 55% of world’s HCC cases occur in the country [4]. Surgical resection provides an opportunity for cure, but frequent recurrence after surgery remains the major obstacle to long-term survival [5]. It is estimated that approximately 70% of patients will relapse within 5 years after surgery and more than 80% of postoperative recurrence occurs in the remnant liver [6], which can be either intrahepatic metastasis from the primary tumor or de novo multicentric tumors. Typically, recurrence in HCC follows a 2-peak distribution: the first peak, usually within 2 years after resection, is mostly related to true metastatic spread (i.e., early recurrence), whereas the second peak mainly results from de novo tumors as a consequence of the carcinogenic cirrhosis (i.e., late recurrence) [7]. Vascular invasion (macroscopic and microscopic) is the strongest predictor of recurrence although other factors such as tumor size, number of nodules, α-fetoprotein (AFP) levels, degree of differentiation, and satellite lesions are also associated with recurrence [6]. Unfortunately, microvascular invasion and satellites can be assessed only with the full pathologic specimen, which reduces the odds for an accurate preoperative prediction of HCC recurrence. In addition to cancer, another life-threatening condition (i.e., cirrhosis) is present in more than 80% of patients with HCC, which renders prognostic prediction a major challenge. Some clinical-based staging systems, especially the widely accepted Barcelona Clinic Liver Cancer (BCLC) algorithm [8], establish a road map for routine clinical decision-making. However, these systems fail to provide molecular information, which can complement the portrait of prognosis in complex solid neoplasms. Therefore, elucidating the molecular mechanisms underlying recurrence is essential for identifying accurate predictive biomarkers and developing effective therapeutic modalities.

To date, some cancer cell-oriented predictive systems are neither superior to morphological classification nor display any overlapping predictor genes, and they include few disease-related genes [9,10]. It seems that high levels of HBV replication contribute to the recurrence and poor prognosis of HCC, which is linked to inflammatory cell infiltration. Thus, the liver inflammatory response and the whole-body immune status can largely influence the biological behavior of HCC. Peripheral blood mononuclear cells (PBMCs), the most common immune cell subsets, are transported throughout the entire body. Some PBMC genes may reflect behavior, especially that of HBV-related HCC, which is closely related to the inflammatory response. In addition, it has been reported that some related signals play crucial roles in cancer and inflammation by controlling the expression of certain cytokines [11]. These cytokines, such as IL-6, are produced by lymphocytes in liver and peripheral blood. As a result, some characteristics of genes in PBMCs may be related to the pathogenesis and progression of HCC.

In this study, the whole-genome Affymetrix GeneChip® Human Genome U133 Plus 2.0 Array was applied to define a comprehensive copy number profile in PBMCs that predicts HCC recurrence. The differentially expressed mRNAs were then selected, validated, and subjected to gene ontological (GO) and pathway analysis. The target genes predominating in the gene regulatory networks were further investigated in an attempt to provide better understanding of the biological features of HCC recurrence. Moreover, to ensure that the signature reflecting the profile of recurrence, we simultaneously tested the potential biomarkers from 2 different kinds of patient samples, including PBMCs and cancerous tissues.

## Results

### Identification of recurrence-associated genes in HCC

To indentify candidate genes related to HCC recurrence, a microarray-based gene expression profiling was analyzed. In all, mRNA derived from 6 HCC cases (3 cases with recurrence and 3 without recurrence) were subjected to genome-wide analysis. The results showed that a set of 615 mRNAs were differentially expressed in HCC patients with recurrence, among which 331 mRNAs increased and 284 mRNAs decreased, compared with those without recurrence (Additional file 1: Figure S1).

**Figure 1:**
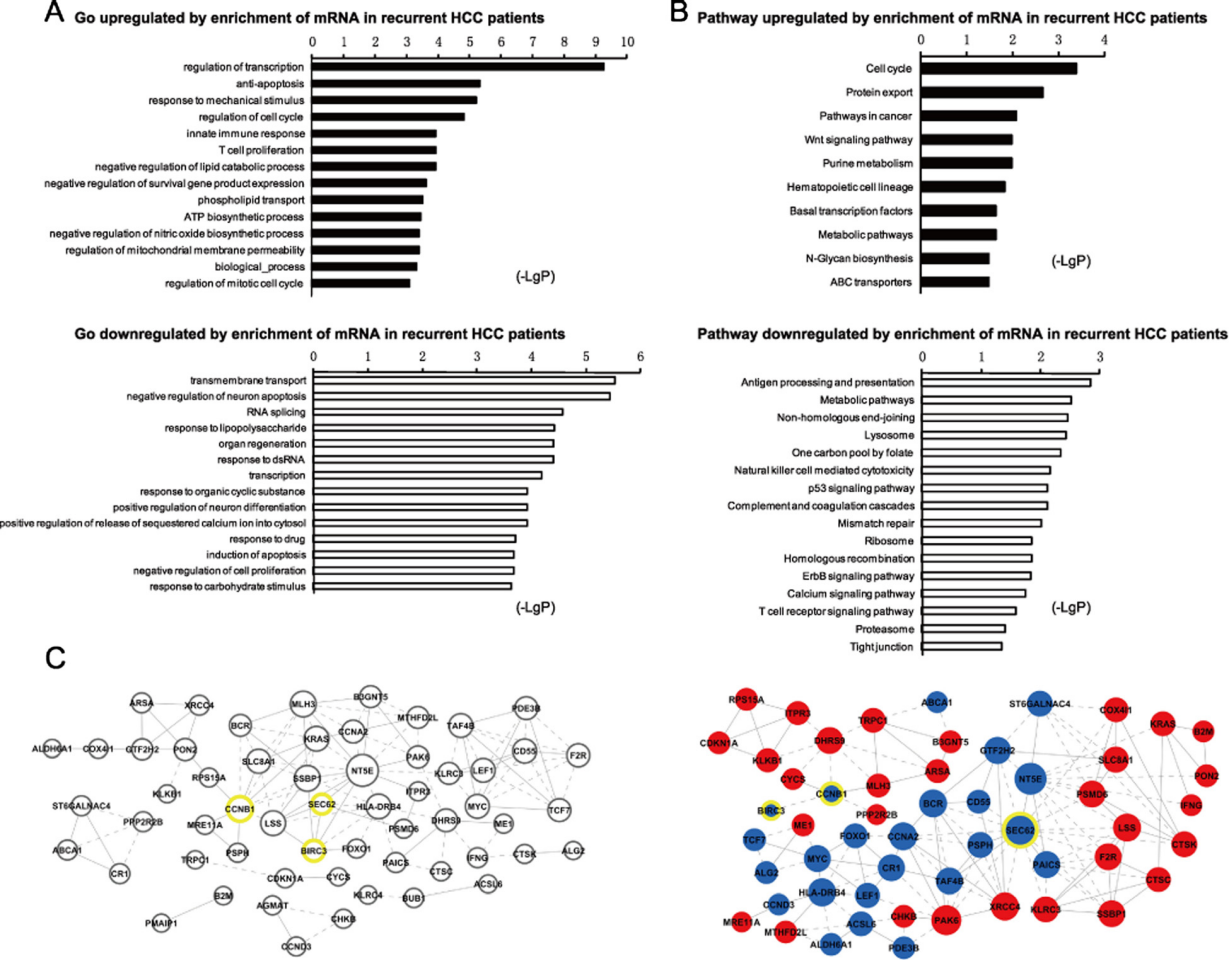
**GO and pathway ehrichment and interaction network analyses based on the set of 615 differentially expressed genes in the recurrent HCC and non-recurrent HCC samples**. **A.** GO category based on the biological process for differentially expressed genes. (Upper) The significant GO category for the upregulated genes. (Below) The significant GO category for downregulated genes. LgP is the base-10 logarithm of the p value. **B.** KEGG pathway analysis for the differentially expressed genes. (Upper) The significant pathway for the upregulated genes. (Below) The significant pathway for the downregulated genes. **C.** Interaction network analysis of the 615 genes. The 615 altered genes were connected in a network based on prior known protein-protein interactions and signaling pathways. Blue, upregulated genes. Red, downregulated genes. The *CCNB1*, *SEC62* and *BIRC3* genes had the highest *DiffK*(*i*) values; therefore, they might be of great importance to HCC recurrence in these patients.

To further determine mRNAs involved in the cellular behavior and signaling pathways, we conducted a GO enrichment analysis. These 615 mRNAs were enriched for cancer-dominant functions, such as anti-apoptosis, cell cycle regulation, and transmembrane transport (Figure 1A). The Kyoto Encyclopedia of Genes and Genomes (KEGG) functional analysis of mRNAs revealed that 10 signaling pathways were upregulated, whereas 16 were downregulated (Figure 1B). Many of these signaling pathways, such as antigen processing and presentation, cell cycle, and protein export, have been demonstrated to participate in the activation of HCCs. Among these differentially regulated signaling pathways, the cell cycle appeared to be the most enriched pathway. A similar phenomenon was observed in the GO analysis.

Furthermore, we constructed a co-expression network using the k-core algorithm to determine which gene(s) may play pivotal roles in the recurrence of HCC according to their GO and pathway terms (Figure 1C). Some critical genes were located in these modules, including cycling B1 (*CCNB1*), SEC62 homolog (*S. cerevisiae*) (*SEC62*), and baculoviral IAP repeat-containing 3 (*BIRC3*) (Figure 1D), which had the highest *DiffK*(*i*) values, suggesting that they probably play important roles in the pathogenesis of HCC recurrence.

To confirm the results of microarray analysis, we examined the mRNA expressions of these 3 genes using quantitative real-time polymerase chain reaction (RT-PCR; Additional file 2: Figure S2).

### Elevated expression of cyclin B1, Sec62, and Birc3 in HCC patients with recurrence

**Figure 2:**
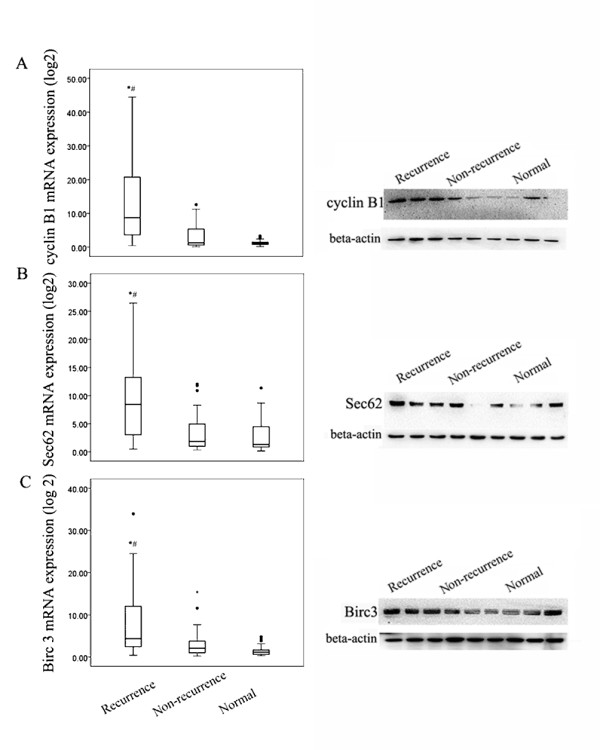
**High expression levels of cyclin B1, Sec62, and Birc3 in the patients with recurrent HCC. A.** The mRNA (left) and protein (right) expression of cyclin B1 was significantly higher in the patients with recurrent HCC than in those with non-recurrent HCC and in the healthy controls (*p* < 0.001). However, no difference was found between the non-recurrence and healthy groups (*p* > 0.05). **B.** The mRNA (left) and protein (right) expression of Sec62 were significantly higher in the patients with recurrent HCC than those with non-recurrent HCC and in the healthy controls (*p* < 0.001). However, no difference was found between the non-recurrence and healthy groups (*p* > 0.05). **C.** The mRNA (left) and protein (right) expression levels of Birc3 were significantly higher in the patients with recurrent HCC than in those with non-recurrent HCC and in the healthy controls (*p* < 0.001). However, no difference was found between the non-recurrence and healthy groups (*p* > 0.05). * Compared with non-recurrence group. ^#^ Compared with the healthy group. The mRNA expression levels were quantified using quantitative PCR. GAPDH was used as the endogenous control for the mRNA levels. The protein levels were examined by western blotting. β-actin was used as the endogenous control for the protein levels.

To explore whether cyclin B1, Sec62, and Birc3 are key molecular markers in predicting HCC recurrence, we measured the expression levels of these 3 proteins in 80 HCC samples from HCC cases and 30 samples from healthy subjects. Of the 35 recurrent HCC samples, we found that the transcriptional and protein expressions of cyclin B1, Sec62, and Birc3 in the PBMCs were significantly higher than those in the non-recurrent and normal samples (*p* < 0.001, *p* < 0.001, and *p* < 0.001, respectively, Figure 2A-C). However, no significant difference was found between the non-recurrent and normal samples (*p* = 0.581, *p* = 0.191, and *p* = 0.076, respectively, Figure 2A-C).

**Figure 3:**
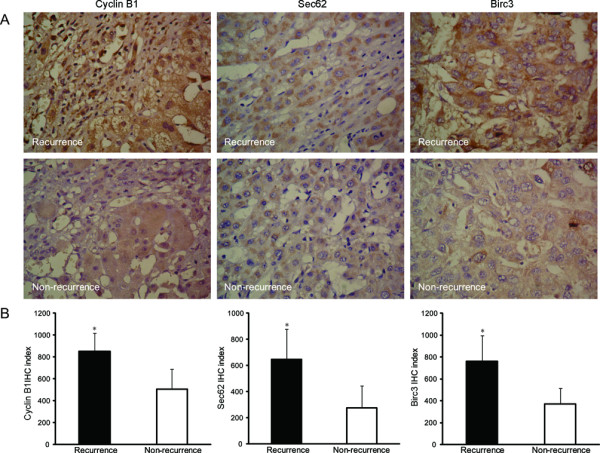
**High expression levels of cyclin B1, Sec62, and Birc3 in the livers of the patients with recurrent HCC. A.** Immunohistochemestry stain for cyclin B1, Sec62, and Birc3 expression (original magnification × 400). Strong expression of these proteins was obserced in the livers of the patients with recurrent HCC. By contrast, the expression of these proteins was apparently decreased in the patients with non-recurrent HCC. **B.** cyclin B1, Sec62, and Birc3 immunohistochemical indices. The cyclin B1, Sec62, and Birc3 immunohistochemical indices were significantly higher in the recurrent HCC samples than in the non-recurrent HCC samples. (*p* < 0.05). The data are mean ± SD values. * *p* < 0.05 compared with the non-recurrence group.

To further determine the clinicopathologic significance of cyclin B1, Sec62, and Birc3 in HCC, immunohistochemical analysis was performed from 35 recurrent tissues and 45 non-recurrent ones. As shown in Figure 3A and B, the protein levels of cyclin B1, Sec62, and Birc3 were substaintialy higher in the recurrent tissues than those in the non-recurrent samples. Importantly, the results were consistent with the transcriptional and protein results in PBMCs, which suggested that elevated expression of cyclin B1, Sec62, and Birc3 may be critical to the recurrence of HCC.

### Association of cyclin B1, Sec62, and Birc3 expression with HCC recurrence

From ROC analysis, we found that 80%, 65.7%, and 54.2% of the patients with recurrent HCC exhibited highly expressed cyclin B1, Sec62, and Birc3, respectively. By contrast, most of non-recurrent HCC patients had low expression levels of these proteins (*p* < 0.001, *p* < 0.001, and *p* = 0.001, respectively, based on cut off values that discriminate recurrent from non-recurrent samples).

**Figure 4:**
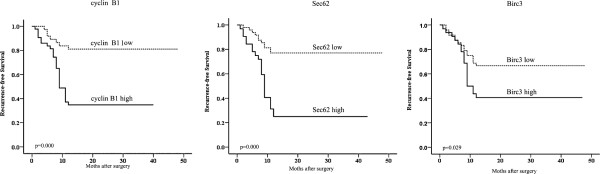
**The clinical significance of cyclin B1, Sec62, and Birc3 expressions in the patients with HCC surgery.** Kaplan-Meier curves were used to estimate the recurrence-free survival rates according to the expression levels of cyclin B1, Sec62, and Birc3 in the 80 HCC patients who underwent surgery. Cyclin B1 (left), Sec62 (middle), and Birc3 (right).

Upon clinicopathological correlation analysis, segregation of patients into the high expression of cyclin B1/Sec62/Birc3 and low expression revealed no significant correlations with any single clinicopathological features, including age, sex, AFP, histopathological grading, tumor number, or liver cirrhosis. Furthermore, we investigated the correlation of cyclin B1, Sec62, and Birc3 expressions with survival by univariate and multivariate survival analysis. We found that overexpression of cyclin B1, Sec62, and Birc3 was correlated with earlier recurrence in HCC patients who underwent surgical resection (*p* < 0.001, *p* < 0.001, and *p* = 0.029, respectively, Figure 4).

Finally, based on the univariate analysis, we further determined the independent prognostic factors for predicting HCC recurrence (Table 1). The analysis revealed that clinicopathological features provided significant predictive value for recurrence, including preoperative AFP levels (*p* < 0.001), tumor number (*p* = 0.002), and liver cirrhosis (*p* = 0.02), which were consistent with previous results [12-14], suggesting that the selected samples in this study represent the characteristics of HCC patients.

**Table 1:** Univariate analyses of predictors of recurrence in HCC patients

**Variables**	**HCC patients (n = 80)**		
	**recurrence**	**non-recurrence**	*p*-value *
Tumor Number			0.002
solitary	14	33	
multiple	21	12	
Liver cirrhosis			0.020
yes	18	34	
no	17	11	
Differentiation			0.252
I-II	16	27	
III-IV	19	18	
AFP(ng/ml)			0.001
≤400	11	33	
>400	24	12	
Cyclin B1 mRNA			0.001
high	28	15	
low	7	30	
Sec62 mRNA			0.001
high	23	8	
low	12	37	
Birc3 mRNA			0.029
high	19	11	
low	16	34	

* Statistical analyses were conducted by Kaplan-Meier method (log rank-test).

The following Cox multivariate analysis revealed that cyclin B1 or Sec62 overexpression could be a novel independent prognostic factor for recurrence-free survival after surgery (cyclin B1: hazard ratio [HR], 4.762; 95% confidence interval [CI], 1.764–12.856; *p* = 0.002; SEC62: HR, 2.647; 95% CI, 1.181–6.057; *p* = 0.018, Table 2).

**Table 2:** Cyclin B1, Sec62 mRNA expression in HCC were independent Predictive factors for recurrence

**Variable**	**HR (95%CI)**	*p*-value *
AFP Level		
>400 ng/ml vs ≤ 400 ng/ml	3.124 (1.294–7.542)	0.011
cyclin B1 expression		
high vs low	4.762 (1.764–12.856)	0.002
Sec62 expression		
high vs low	2.674 (1.181–6.057)	0.018

HR: risk ratio; 95% CI: 95% confidence interval; * Cox proportional hazards regression.

Taken together, the above findings indicate that cyclin B1 and Sec62 are important predictors of metastatic recurrence of HCC in patients after surgery, which may influence overall survival of patients.

## Discussion

In the present study, we found cyclin B1, Sec62, and Birc3 were aberrantly expressed proteins in HCC patients. Highly expressed cyclin B1, Sec62, and Birc3 were associated with significantly reduced recurrence-free survival, and cyclin B1 and Sec62 were independent prognostic factors in this cohort. To our knowledge, this is the first detailed systematic investigation of the expression pattern in PBMCs and the roles of these 3 proteins, especially Sec62, in HCC recurrence.

The current consensus is that surgery is one of the most important treatment options for patients with HCC. However, tumor recurrence remains one of the major challenges for those postoperative patients. Although increasing numbers of genes have been indentified, the molecular mechanism of HCC metastasis and recurrence are not fully understood. Based on the results of microarray analysis, cyclin B1, Sec62, Birc3 were chosen for subsequent research. Cyclin B1 is known to regulate the G2/M transition in the cell cycle. Recent studies have demonstrated aberrant expression of cyclin B1 in several malignant cancers, including breast cancer [15], esophageal squamous cell carcinoma [16], non-small cell carcinoma [17], gastric cancer, and hepatocellular carcinoma [18,19]. But it remains unclear how cyclin B1 overexpression is involved in oncogenesis and tumor progression. Previous study demonstrated that cyclin B1 act as a promising prognostic and therapeutic target for HCC [20]. However, Chae [21] reported that the expression of cyclin B1 had no influence on the survival of patients with breast cancer. In the present study, elevated expressed cyclin B1 was found in the patients with recurrent HCC, contrary to that in non-recurrent patients and healthy volunteers. Moreover, there was no significant difference in cyclin B1 expression between the patients with non-recurrent HCC and healthy subjects. Through the univariate analysis, cyclin B1 expression was identified as an independent risk factor for recurrence in HCC patients after surgery. This discrepancy might be due to dissimilar expression of cyclin B1 in different tumor types.

Similar results were observed for Sec62, which is a member of the protein translocation apparatus in the endoplasmic reticulum membrane. Previous studies demonstrated the amplification and overexpression of Sec62 in prostate cancer cell lines, and described *SEC62* as a potential target gene in prostate cancer [22]. Overproduction of Sec62 is also observed in other tumors, primarily in tumors of the lung and thyroid [23]. In our study, it seems that Sec62 plays a significant role in HCC recurrence. Sec62 overexpression was found in the patients with recurrent HCC. Importantly, Sec62 was an independent risk factor for recurrence in HCC patients after surgery as evidenced by univariate analysis.

Although the expression of Birc3 was significantly higher in the recurrent HCC samples than that in the non-recurrent HCC and normal samples, a specific independent role in predicting HCC recurrence was not identified for Birc3. Consistently, DNA amplifications of Birc2 and Birc3 have been observed in mouse liver and human lung cancers [24,25], liver carcinoma [24], oral squamous cell carcinoma [26,27], medulloblastoma [28], glioblastoma [29], and pancreatic cancer [30]. The exact role of Birc3 in HCC must be verified through a larger prospective study.

In recent years, studies on malignant tumors has primarily focused on cell proliferation, migration, and apoptosis. Cyclin B1, Sec62, and Birc3, chosen in this study according to our microarray analysis, likely play important roles in cell proliferation and migration. They can exert a tumor-promoting effect on HCC by regulating cell cycle and protein translocation. In contrast to previous studies using only HCC tissues, we examined PBMCs and tumor tissues in the present study. Interestingly, the results obtained in PBMCs were consistent with those of the tumor tissues by immunohistochemical analysis for. As a result, elevated cyclin B1 and Sec62 expression in PBMCs had a significantly negative prognostic value in terms of recurrence-free survival, which hints the potential use of these molecular markers to predict the risk of tumor recurrence after surgery and to act as therapeutic targets to reduce tumor recurrence and improve clinical therapies.

The contribution of HBV to the current findings must be mentioned. China is one of the highest prevalent areas of HCC, mainly because chronic hepatitis B carriers account for more than 10% of the Chinese population [31]. Over 85% of patients with HCC have HBV infection in China [32]. At present, the studied population almost unavoidably consisted of patients with HBV-associated HCC because of the special situation in China. The induction of apoptosis and stimulation of cell cycle by the HBV X protein has been reported [33,34]. The analysis of cyclin B1, Sec62, and Birc3 expressions in HCC patients with other etiological backgrounds may be very useful to ascertain the real predictive value of cyclin B1 and Sec62 for HCC recurrence.

Despite the important roles of cyclin B1 and Sec62 in tumor recurrence and their predictive implications, this study should be viewed as a hypothesis-generating study. Prospective and animal studies are needed to confirm our findings and clarify the biological effects of these proteins in more detail.

## Conclusions

This study demonstrates a significant association between high cyclin B1 and Sec62 expression levels and HCC recurrence, indentifying cyclin B1 and Sec62 as predictors of HCC recurrence. More importantly, their expressions in the PBMCs were consistent with those in the HCC tissues. These findings also suggest that cyclin B1 and Sec62 might be potential molecular targets to reduce tumor recurrence.

## Methods

### Cytokines and reagents

The RT reagent kit was purchased from Takara (Dalian, China). The SYBR Green Real-Time PCR Master Mix kit was purchased from Toyobo (Osaka, Japan). Cyclin B1 (V152) mouse mAb and Birc3 (58 C7) rabbit mAb were purchased from Cell Signaling Technology (Danvers, MA). Sec62 (N-15) pAB sc-12324 was purchased from Santa Cruz Biotechnology (Santa cruz, CA). Lymphocyte separation medium (LSM 1077) was purchased from PAA (MA). Trizol reagent (U.S.patent No. 5,346,994) was purchased from Invitrogen (Carlsbad, CA).

### Patient characteristics

A total of 80 HCC patients with early stage (BCLC A) diease who underwent surgery between 2007 and 2011 in the Changhai Hospital were enrolled in the present study. All the subjects provided written informed consent for the use of their blood samples and HCC tissues in accordance with the Declaration of Helsinki, and the study protocol was approved by our institutional review board. HCC was diagnosed either before or after surgery and confirmed by histopathological examination, and complete clinical and laboratory data were available before surgery and during follow-up. The characteristics of the HCC patients, including age, sex, tumor size, portal vein tumor thrombi (PVTT), BCLC, Child-Pugh, cirrhosis and preoperative AFP levels, are shown in Table 3. Tumor differentiation was graded by the Edmondson-Steiner grading system. The eligibility criteria for the patients studied are as follows: (a) HCC diagnosed either before or after surgery (as an incidental finding) and confirmed by histopathological examination; (b) Han Chinese ethnicity; (c) the availability of AFP level, histopathologic grading, tumor size, and tumor number data before surgery and during follow-up; (d) HBV-positivity and hepatitis C virus negativity; and (e) no preoperative adjuvant antineoplastic therapy. The follow-up course and diagnostic criteria of recurrence have been described previously [12]. The median duration of follow-up was 39.0 months. Using ≤12 and ≥36 months as the cutoffs, the patients were divided into recurrence and non-recurrence groups. In EDTA-K2 tubes, 10 mL of anticoagulated blood were collected from HCC patients between 6:30 and 7:00 am when they were admitted to the Changhai Hospital. All the blood samples were used for PBMC isolation. Microarray experiments were performed at the Shanghaibio Corporation (National Engineering Center for Biochip in Shanghai, China) using the Affymetrix GeneChip® Human Genome U133 Plus 2.0 Array (Affymetrix, Santa Clara, CA, USA). The RNA for real-time PCR and the protein for western blotting were prepared as described previously (primers are shown in Additional file 3: Table S1) [35,36].

**Table 3:** Clinicopathologic charateristics in HCC patients

**Variables**	**HCC patients(n = 80)**		
	**non-recurrence**	**recurrence**	*p*-value *
NO. of HCC	45	35	
Age(years)			0.260
≤50	20	20	
>50	25	15	
sex			0.798
male	39	31	
female	6	4	
Tumor number			0.003
single	33	14	
mutiple	12	21	
Tumor size			1.000
≤5 cm	45	35	
Liver cirrhosis			0.025
yes	34	18	
no	11	17	
Differentiation			0.204
I-II	27	16	
III-IV	18	19	
AFP(ng/ml)			0.001
≤400	33	11	
>400	12	24	
PVTT			1.000
negative	45	35	
Child-pugh			1.000
A	45	35	
BCLC stage			1.000
A	45	35	

* *p*-value calculated using chi-square or Fishier exact test.

AFP, alpha-fetoprotein; BCLC, Barcelona clinc liver cancer.

### Differentially expressed probe sets

For identifying significant probe sets, the random variance model (RVM, which is commonly used for comparisons of more than 2 groups) *T* test was applied to the entire probe sets [37]. Values of p < 0.05 and false discovery rates < 10% were considered statistically significant.

### Hierarchical cluster, GO, pathway, and GeneRel net (co-expression network) analyses

To ascertain whether differentially expressed genes among the groups were selected correctly, unsupervised hierarchical cluster analysis was performed using 615 identified genes. The significant genes in each unique pattern were subjected to a GO analysis (http://www.geneontology.org/). The GO analysis was applied to organize the genes into hierarchical categories and uncover the co-expression network according to biological process and molecular function. The co-expression network of gene interaction, representing the critical mRNAs and their targets, was established according to mRNA expression [38]. Mean-while, the significant genes in unique patterns were subjected to a KEGG analysis (http://www.genome.jp/kegg/), which was performed on the basis of scoring. In detail, a 2-sided Fisher’ exact test and a chi-square test were used to classify the enrichment (*R*_e_) of the GO and pathway categories. The enrichment (*R*_e_) was calculated as follows:

(1) Re=nfnNfN

where *n*_f_ and *n* represent the numbers of target genes and total genes, respectively, in the particular GO or pathway category and *N*_f_ and *N* represents the number of genes among the entire differential corresponding target genes and the total number of genes in the GO or pathway categories, respectively. We used gene co-expression networks to elucidate the interactions among the genes. Gene co-expression Networks were built according to the normalized signal intensity of specific expression genes. For each pair of genes, we calculated the Pearson correlation and chose the significant correlation pairs to construct the network [39]. Within the network analysis, degree centrality is the simplest and most important measure to determine the relative importance of a gene within a network [40]. The genes potentially vital to HCC recurrence were chosen on the basis of measure differential connections between 2 networks. For the *i*th gene, we denoted the whole-network connectivity in networks 1 and 2 by *k*_1_(*i*) and *k*_2_(*i*), respectively. To facilitate the comparison between the connectivity measures of each network, we divided each gene connectivity by the maximum network connectivity as follows: ${\text{K}}_{1}(i)=\frac{k_{1}(i)}{\max({\text{k}}_{1})}$ and ${\text{K}}_{2}(i)=\frac{k_{2}(i)}{\max({\text{k}}_{2})}$.Next, we defined a measure of differential connectivity as *DiffK*(*i*) = *K*_1_(*i*) – *K*_2_(*i*). The significance of a gene increased as the value of *DiffK*(*i*) increased [41].

### Immunohistochemistry

Immunohistochemical staining was performed as described previously [42]. The expression levels of cyclin B1, Sec62, and Birc3 were calculated by the number of positive cells per 1000 hepatocytes counted, which was defined as LI. For cyclin B1 staining, brown-stained nucleus was scored as positive. For Sec62 staining, a brown-stained plasmalemma was scored as positive. For Birc3 staining, brow staining in the cytoplasm was scored as positive. The cyclin B1, Sec62, Birc3 expressions were quantitatively evaluated using an Olympus BH2 microscope with a computer-aided image analysis system (Qiu Wei Inc, Shanghai, China). The digital images were archived by a digital camera (Nikon 4500, Tokyo, Japan). The positive area and optical density (OD) of cyclin B1, Sec62, or Birc3-positive cells were determined by measuring 3 randomly selected microscopic fields (25, 9, 10) for each slide. The immunohistochemical index was defined as the mean integral optical density (AIOD; AIOD = positive area × OD/total area).

### Data analyses

Statistical analyses were performed using SPSS version 15.0 (SPSS, Chicago, IL). The Kruskal-Wallis and Mann–Whitney *U* nonparametric tests were used for the statistical comparison of the variables between the investigated groups. The predictive accuracy was calculated using the ROC. The probability of recurrence-free survival was analyzed by the Kaplan-Meier method, and the differences between the groups were estimated by the log-rank test. Independent prognostic indicators were assessed by multivariate analysis using Cox’s proportional hazard model. The Pearson chi-square test or Fisher exact test was used to compare qualitative variables, and quantitative variables using the Spearman correlation test. *p* < 0.05 (2-tailed) was considered significant.

## Abbreviations

HCC, Hepatocellular Carcinoma; HR, Hazard Ratio; SEC62, SEC62 homolog(S.cerevisiae); BIRC3, Baculoviral IAP repeat-Containing3; AFP, α-fetoprotein; BCLC, Barcelona Clinic Liver Cancer; PBMC, Peripheral Blood Mononuclear Cells; ROC, Receiver Operating Characteristic Curve; GAPDH, Glyceraldehyde-3-phosphate Dehydrogenase; RT-RCR, Quantitative Real-time Polymerase Chain Reaction; HBV, Hepatitis B virus; KEGG, Kyoto Encyclopedia of Genes and Genomes.

## Competing interests

The authors declare that they have no competing interests.

## Authors’ contributions

Li W and Juan Du made the microarrays, performed the real-time, western-blot and immunohistochemical staining, collected the clinical data and contributed to the writing of the manuscript. Qinghui Z analyzed the statistical data and participated in writing the manuscript. Binbin C and Jun L participated in collecting the clinical data. Changquan L and Denghai Z participated in designing and coordinating the study, and in writing the manuscript. All of the authors read and approved the final manuscript.

## Supplementary Material

Additional file 1 **Figure S1.** Unsupervised hierarchical clustering based on the set of 615 differentially expressed genes in response to recurrent HCC patients and non-recurrent samples. The relative gene log 2 expression changes are expressed by a color gradient intensity scale, as shown in the upside. Green color indicates down-regulation, and red color indicates up-regulation of gene expression. Each row represents a separate sample and each column a single gene. Click here for file

Additional file 2 **Figure S2.** The mRNA levels of cyclin B1, Sec62 and Birc3 in 6 HCC patients were analysed by RT-PCR. To validate the microarray analysis findings, we analyzed their mRNA expression using real-time PCR in 6 samples. The expression of cyclin B1, Sec62 and Birc3 in recurrence HCC Patients were significantly higher than non-reucrrence samples (*p* < 0.05). Cyclin B1 (left), Sec62 (median), and Birc3 (right). * compared with non-recurrence group. Click here for file

Additional file 3 **Table S1.** Primers for selected genes analyzed by RT-PCR. Click here for file
