# Supplementary figures and images for: Identification of cyclin B1 and Sec62 as biomarkers for recurrence in patients with HBV-related hepatocellular carcinoma after surgical resection
Source: Mol Cancer. 2012 Jun 8;11:39. doi: 10.1186/1476-4598-11-39 (PMC3439291; doi:10.1186/1476-4598-11-39)

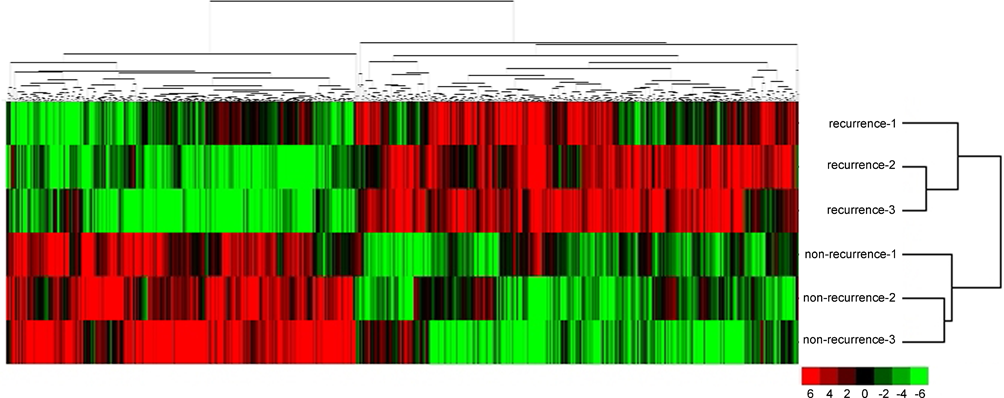

Supplement: Additional file 1 — Figure S1. Unsupervised hierarchical clustering based on the set of 615 differentially expressed genes in response to recurrent HCC patients and non-recurrent samples. The relative gene log 2 expression changes are expressed by a color gradient intensity scale, as shown in the upside. Green color indicates down-regulation, and red color indicates up-regulation of gene expression. Each row represents a separate sample and each column a single gene. [file 1476-4598-11-39-S1.tiff]

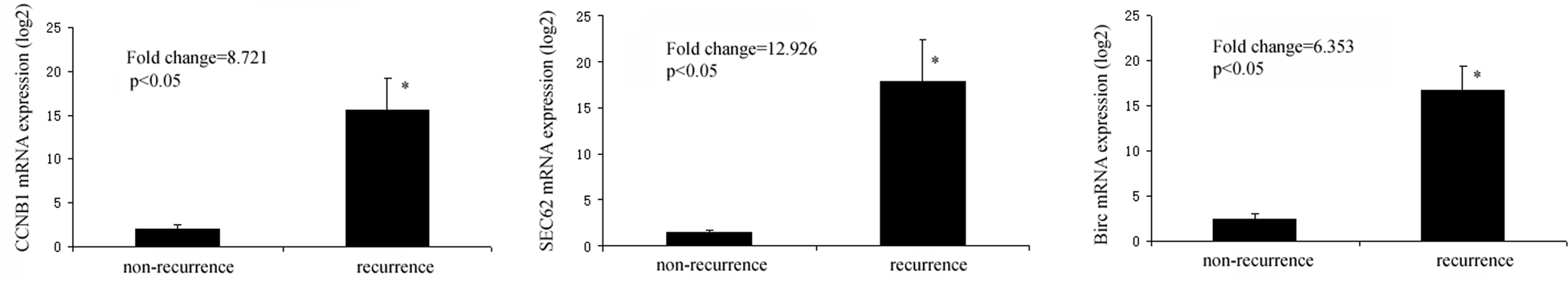

Supplement: Additional file 2 — Figure S2. The mRNA levels of cyclin B1, Sec62 and Birc3 in 6 HCC patients were analysed by RT-PCR. To validate the microarray analysis findings, we analyzed their mRNA expression using real-time PCR in 6 samples. The expression of cyclin B1, Sec62 and Birc3 in recurrence HCC Patients were significantly higher than non-reucrrence samples (p < 0.05). Cyclin B1 (left), Sec62 (median), and Birc3 (right). * compared with non-recurrence group. [file 1476-4598-11-39-S2.tiff]
