# Supplementary material for: Identification of cyclin B1 and Sec62 as biomarkers for recurrence in patients with HBV-related hepatocellular carcinoma after surgical resection
Source: Mol Cancer. 2012 Jun 8;11:39. doi: 10.1186/1476-4598-11-39 (PMC3439291; doi:10.1186/1476-4598-11-39)
Supplement: Additional file 3 — Table S1. Primers for selected genes analyzed by RT-PCR. [file 1476-4598-11-39-S3.doc]

**Table S1** Primers for selected genes analyzed by RT-PCR

| Symbol | Gene description | Primersequence(5’-3’) | GeneBank accession | Product (bp) |
| --- | --- | --- | --- | --- |
| *CCNB1* | Cyclin B1 | GAAGATCAACATGGCAGGCG | NM-031966 | 131 |
|  |  | GCATTTTGGCCTGCAGTTGT |  |  |
| *SEC62* | SEC62 homolog (S. cerevisiae) | CAAAGCTGACCCGAATGGAC | NM-003262 | 121 |
|  |  | AGAGCCGAGAATAGGCACCC |  |  |
| *BIRC3* | baculoviral IAP repeat-containing 3 | CCATTGACTTTTCTGTCGCCA | NM-001165 | 125 |
|  |  | GCATTATCCTTCGGTTCCCAA |  |  |
| hsaGAPDH |  | TGTTGCCATCAATGACCCCTT |  | 201 |
|  |  | CTCCACGACGTACTCAGCG |  |  |
